# Anti-aquaporin-4 immune complex stimulates complement-dependent Th17 cytokine release in neuromyelitis optica spectrum disorders

**DOI:** 10.1038/s41598-024-53661-5

**Published:** 2024-02-07

**Authors:** Shuhei Nishiyama, Jin Myong Seok, Amy E. Wright, Itay Lotan, Takahisa Mikami, Natalia C. Drosu, Natasha Bobrowski-Khoury, Monique R. Anderson, Philippe A. Bilodeau, Patrick Schindler, Friedemann Paul, Masashi Aoki, Michael R. Yeaman, Michael Levy, The Guthy-Jackson Charitable Foundation CIRCLES Study Group, Jacinta M. Behne, Megan K. Behne, Jeffrey L. Bennett, Terrence F. Blaschke, Tanuja Chitnis, Lawrence J. Cook, Michael Levy, Sarah M. Planchon, Pavle Repovic, Claire S. Riley, Terry J. Smith, Anthony Traboulsee, Michael R. Yeaman

**Affiliations:** 1 Department of Neurology, Massachusetts General Hospital, Boston, MA USA; 2 Harvard Medical School, Boston, MA USA; 3 Department of Neurology, Tohoku University Graduate School of Medicine, Sendai, Miyagi Japan; 4 Department of Neurology, Massachusetts General Hospital, 65 Landsdowne, Lab 500, Cambridge, MA 02139 USA; 5 Experimental and Clinical Research Center, Max Delbrück Center for Molecular Medicine and Charité – Universitätsmedizin Berlin, corporate member of Freie Universität Berlin and Humboldt-Universität zu Berlin, Berlin, Germany; 6 Geffen School of Medicine at UCLA, Los Angeles, CA USA; 7 Division of Molecular Medicine, David Geffen School of Medicine at UCLA, Institute for Infection and Immunity, Harbor-UCLA Medical Center, Lundquist Institute at Harbor-UCLA Medical Center, Torrance, CA USA; 8 The Guthy-Jackson Charitable Foundation, Beverly Hills, CA USA; 9 Departments of Neurology and Ophthalmology, University of Colorado School of Medicine, Aurora, CO USA; 10 Stanford University, Stanford, CA USA; 11 Department of Neurology, Brigham and Women’s Hospital, Harvard Medical School, Boston, MA USA; 12 University of Utah, Salt Lake City, UT USA; 13 Mellen Center for MS Treatment and Research, Neurological Institute, Cleveland Clinic, Cleveland, OH USA; 14 Swedish Medical Center, Seattle, WA USA; 15 Department of Neurology, Columbia University Medical Center, New York, NY USA; 16 University of Michigan Medical School, Ann Arbor, MI USA; 17 Department of Medicine & Neurology, University of British Columbia, Vancouver, BC Canada

**Keywords:** Autoimmunity, Neurology

## Abstract

Proinflammatory cytokines, such as (IL: interleukin) IL-6 and IL-17A, and complement fixation are critical in the immunopathogenesis of neuromyelitis optica spectrum disorders (NMOSD). Blocking the IL-6 receptor or the C5 complement pathway reduces relapse risk. However, the role of interleukin (IL)-6 and complement in aquaporin-4 (AQP4) autoimmunity remains unclear. To investigate the role of the anti-AQP4 immunoglobulin (AQP4-IgG)/AQP4 immunocomplex on the induction and profile of ex vivo cytokine and surface marker expression in peripheral blood mononuclear cells (PBMC) culture. Isolated PBMCs obtained from 18 patients with AQP4-IgG-seropositive-NMOSD (8 treatment-naive, 10 rituximab-treated) or ten healthy controls were cultured with AQP4-immunocomplex with or without complement. Changes in PBMC surface markers and cytokine expression were profiled using flow cytometry and ELISA. PBMCs derived from treatment-naive NMOSD patients stimulated with a complex mixture of serum complement proteins produced significant elevations of IL-17A and IL-6. Rituximab-treated patients also exhibited higher IL-6 but not IL-17A release. IL-6 and IL-17A elevations are not observed without complement. Co-stimulation of PBMCs with AQP4-IgG/AQP4 immunocomplex and complement prompts a Th17-biased response consistent with the inflammatory paradigm observed in NMOSD. A possible inflammation model is proposed via antigen-specific autoreactive peripheral blood cells, including NK/NKT cells.

## Introduction

Neuromyelitis optica spectrum disorder (NMOSD) is an autoimmune inflammatory disease primarily affecting the central nervous system, specifically the optic nerves and spinal cord. Untreated, NMOSD is characterized by recurrent episodes of optic neuritis and longitudinally extensive transverse myelitis, resulting in significant visual impairment and motor dysfunction^1^. NMOSD was previously considered a subtype of multiple sclerosis (MS) but is now recognized as a distinct disorder with its own unique clinical features, diagnostic criteria, and FDA-approved therapeutic agent^1,2^. The presence of aquaporin-4 immunoglobulin G (AQP4-IgG) serum autoantibodies in the majority of NMOSD patients is crucial in distinguishing NMOSD from MS^1,2^.

Aquaporin-4 is a water channel enriched in expression in the central nervous system on astrocyte endfeet, including the optic nerves^3^. The discovery of anti-aquaporin-4 immunoglobulin (AQP4-IgG) antibody has focused attention on its role in antibody-mediated cell cytotoxicity (ADCC) at the astrocyte endfoot in contributing to acute inflammatory responses and secondary demyelination. However, the immunopathogenesis of NMOSD upstream of antibody-mediated CNS injury remains to be elucidated. Production of a pathogenic antibody by plasmablasts implies that one or more mature AQP4-autoreactive B cell clones is stimulated by an AQP4-autoreactive T cell^4^, and that break in tolerance leads to such autoimmunity against AQP4.

Recent studies identified natural killer T cell subsets as potential effectors in the pathogenesis of NMOSD^5^. We have focused on the roles of natural killer (NK) and natural killer T (NKT) cells because of two key clues in the upstream pathogenesis of NMOSD where tolerance is likely to be broken. First, NMOSD is exquisitely responsive to complement inhibition therapy. Eculizumab and ravulizumab reduced the risk of NMOSD relapse by 94% and 98%, respectively, in clinical trials^6,7^. Beyond blocking formation of the membrane attack complex on the surface of astrocytes, complement activation is hypothesized to promote broader CNS inflammatory activity^6^. The upstream target of complement inhibition in NMOSD is not known, but NK and NKT cells are reactive to complement components and their activation in circulation^8^. These cells express both complement C3b/C4b receptor (also known as complement receptor type 1, CR1 or CD35) and C5a receptor (also known as complement component 5a receptor 1, C5aR1 or CD88), the latter of which is significantly overexpressed in people with NMOSD^8^.

Second, when NK and NKT cells are activated by peripheral complement, they upregulate the FCGR3A receptor (CD16) that binds antibody-antigen complexes. The FCGR3A receptor preferentially binds both immobilized or soluble antibody-antigen complexes on dendritic cells and is an extremely efficient mechanism for activating antigen-specific CD4 + T cells^9^. We hypothesized that a similar mechanism may be occurring with NK/NKT cells in NMOSD. In support of this hypothesis, the rs396991 *F176V* polymorphism in the FCGR3A gene affects IgG binding affinity; the V allele of this gene binds IgG (presumably the AQP4-IgG) with greater affinity and is associated with poorer outcomes in NMOSD^10^.

In the current study, we examined the effect of AQP4-IgG/AQP4 immunocomplex on NK and NKT cell phenotypes and cytokine responses in the presence and absence of activated complement in co-culture ex vivo. Among peripheral blood mononuclear cells, we found that NKT cells predominantly bind these immunocomplexes in healthy people and in patients with NMOSD. However, in NMOSD patients, this activity triggers activation of NKT cells predominantly via CD16, leading to a follicular helper cell phenotype and subsequent Th17 biased pro-inflammatory cytokine response, including IL-6, -8, -17A and -23. Importantly, this Th17 polarization response is significantly enhanced by the addition of activated complement. Rituximab treatment in NMOSD attenuates but does not eliminate this Th17-related cytokine release. These results support a model of NMOSD immunopathogenesis in which peripherally activated NKT cells stimulated with AQP4-IgG/AQP4 complexes in the setting of complement fixation promotes a pro-inflammatory process involving T and B cell inflammatory responses in an AQP4-autoantigen specific manner.

## Results

### Variant NKT cells are the first responders to AQP4-immunocomplexes

**Figure 1:**
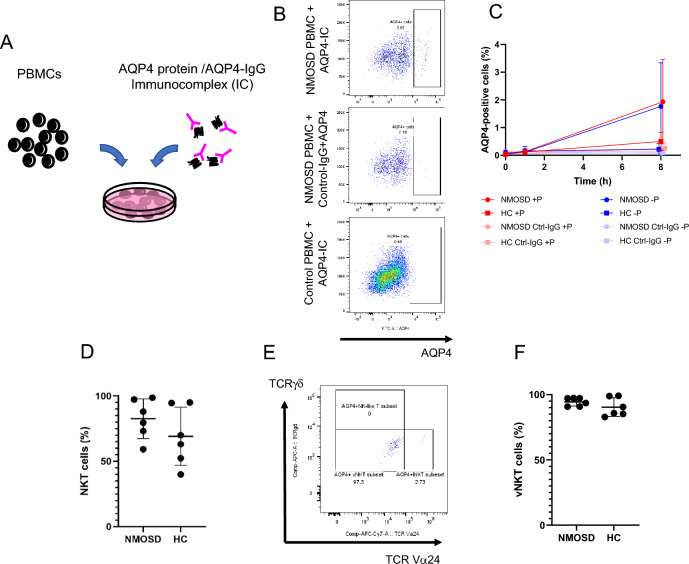
AQP4-immunocomplex kinetics and NKT cells in NMOSD. PBMCs from NMOSD patients and healthy controls (HC) were incubated with AQP4 protein + AQP4-IgG-containing serum (**A**), and analyzed by flow cytometry. After excluding doublet and dead cells, AQP4 positive cells were gated from lymphocytes (**B**). When cultured with the immunocomplexes for 8 h, the group using NMOSD patient-derived PBMCs and AQP4 immunocomplexes detects an AQP4-derived signal ((**B**) top row). In contrast, this signal cannot be detected in the group using control IgG + AQP4 protein ((**B**) middle row) or healthy control PBMCs ((**B**) bottom row). The difference between AQP4-positive lymphocytes with (red dots) or without (blue dots) permeabilization was calculated to detect internalized AQP4. AQP4-positive cells were increased in time-dependent manner (**C**). PBMCs treated with Control-IgG and AQP4 protein did not show AQP4-positive signals. There was little difference between those with and without permeabilization. Majority of the AQP4-positive cells were NKT cells (**D**), and among them, almost all the NKT cell were variant NKT cells (**E**,**F**). PD-1-positive CXCR5-positive subsets were extracted from CD3 + CD56 + CD11b-CD14-CD20-CD66b-NKT cells. + *P* with permeabilization, *-P* without permeabilization, *AQP4-IC* AQP4 protein/AQP4-IgG immunocomplexes, *Ctrl-IgG* Control-IgG, *HC* healthy controls, *RTX* rituximab-treated patients. NMOSD-Naive (n = 8), NMOSD-rituximab treated (n = 10), and healthy controls (n = 10). *p < 0.05; **p < 0.01; ***p < 0.001; ****p < 0.0001.

To determine which immune cells respond to AQP4-IgG and AQP4 protein-immunocomplexes, peripheral blood mononuclear cells (PBMCs) from Healthy Controls (HC) were compared to those from people with AQP4-IgG-positive NMOSD who were treated with rituximab and those who were naïve to all treatments. The AQP4-immunocomplex bound to PBMCs both from NMOSD patients and HC. AQP4-immunocomplex-binding cells increased over time, with 1.930 ± 1.525% of the NMOSD group (n = 3) and 0.500 ± 0.332% of the HC (n = 3) among total live lymphocytes being AQP4-positive after eight-hour incubation (Fig. 1B and C). Analysis of the AQP4-positive cells showed that 82.70 ± 15.12% of the NMOSD (n = 6) and 69.18 ± 22.29% of HC (n = 6) were NKT cells (Fig. 1D). Besides NKT cells, the other cells that became AQP4 positive were T cells (NMOSD 8.752 ± 10.64%; HC 17.24 ± 13.26%) and NK cells (NMOSD 6.623 ± 4.146%; HC 11.29 ± 8.746%, data not shown). Interestingly, most of the AQP4-immunocomplex-binding NKT cells were TCR Vα24-/TCRγδ double negative-variant NKT cells (NMOSD: 94.47 ± 3.147%; HC: 90.38 ± 7.118%, Fig. 1E and F). This subset may also include Mucosal-associated invariant T (MAIT) cells. To identify whether AQP4-immunocomplexes are maintained on the surface or internalized, we compared the difference between the AQP4-positive signals with and without permeabilization. The difference was only 0.2%, with 90% of the immunocomplexes remaining on the membrane after 8-hour incubation (Fig. 1C).

**Figure 2:**
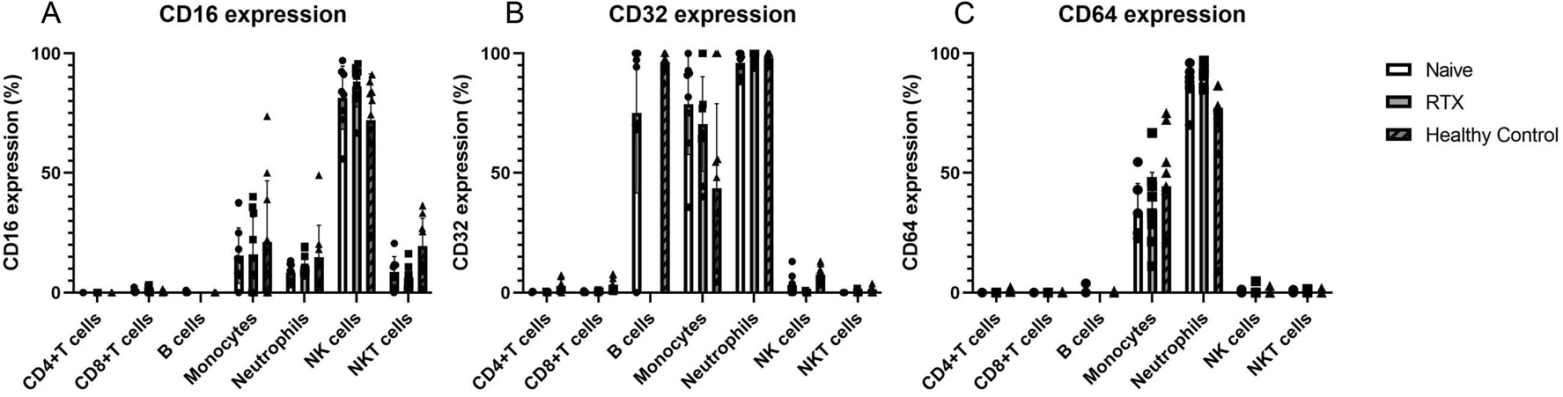
The expression levels of Fc gamma receptors in PBMCs. The expression levels of CD16 (**A**), CD32 (**B**), and CD64 (**C**) were obtained by flow cytometry. NK cells were predominantly CD16 expressing. NKT cells expressed CD16 as well but showed decreased expression in the NMOSD group. Neither CD4 + T cells nor CD8 + T cells expressed Fcɤ receptors. CD32 was positive on B cells, but CD16 and CD64 were not. We couldn’t detect any B cells from all rituximab-treated NMOSD patients. Most of the monocytes had CD32 signals in NMOSD groups. Some monocytes expressed CD16, thought to be either intermediate or non-classical monocytes. Neutrophils were expressing all Fcɤ receptors.

The expression levels of all Fcγ receptors (FcγRs), including CD16, CD32, and CD64, were also examined in all 28 samples used in the study (Fig. 2). Most FcγRs expressed on NK cells were CD16 in all groups, regardless of disease or therapeutic intervention, while CD32 and CD64 expression was minor, suggesting that CD16 is the major Fcγ receptor in NK cells. In contrast, NKT cells from both NMOSD groups expressed less CD16. Other FcγRs were not detected on the surface of NKT cells. As for the other cells examined in this study, CD4/CD8-positive T cells had no FcγRs, while CD32 was significantly expressed in B cells (Note: B cells could not be gated and analyzed in rituximab-treated NMOSD patients). In addition, CD32 expression was high among monocytes, with some CD16-positive intermediate/non-classical monocytes. All FcγRs were highly expressed in neutrophils, regardless of disease or therapeutic intervention. There were no significant differences of CD16, CD32, and CD64 expressions (P = 0.6243, 0.9634, 0.7157, respectively) between treatment-naïve NMOSD patients, rituximab-treated patients, and healthy controls in two-way ANOVA.

### AQP4-immunocomplex and complement stimulation promotes Th17 cytokine release from PBMCs

**Figure 3:**
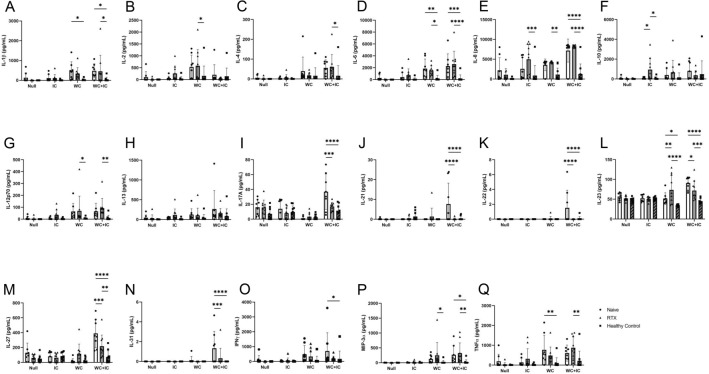
The cytokine levels in media after AQP4-immunocomplex- and whole complement-stimulation of PBMC in vitro. The cytokine levels of IL-1β (**A**), IL-2 (**B**), IL-4 (**C**), IL-6 (**D**), IL-8 (**E**), IL-10 (**F**), IL-12p70 (**G**), IL-13 (**H**), IL-17A (**I**), IL-21 (**J**), IL-22 (**K**), IL-23 (**L**), IL-27 (**M**), IL-31 (**N**), IFNγ (**O**), MIP-3α (**P**), and TNFα (**Q**) were obtained by multiple-ELISA kits. IL-6 and TNFα were significantly elevated in the NMOSD group when complement was added (**E** and **Q**). In contrast, IL-17A and Th17 cytokines were significantly elevated only in the naive NMOSD group when both complement and AQP4 immune complexes were added (**I**–**N**). *IC* AQP4-immunocomplexes, *WC* whole complement, *WC* + *IC* AQP4-immunocomplexes/whole complement treated, *RTX* rituximab-treated NMOSD patients. NMOSD-Naive (n = 8), NMOSD-rituximab treated (n = 10), and healthy controls (n = 10). *p < 0.05; **p < 0.01; ***p < 0.001, ****p < 0.0001.

Cytokine levels in the media were analyzed by ELISA (Fig. 3); proinflammatory cytokines IL-1β and TNF-α, as well as IL-4, IL-6, IL-12p70, and MIP-3α, were significantly elevated in the NMOSD patient group in contrast to HC group after co-stimulation of AQP4 immunocomplexes and complement, regardless of treatment status. Of note, IL-17A and other Th17 cytokines such as IL-21, IL-22, IL-23, IL-27, and IL-31 were significantly upregulated only in the treatment-naïve NMOSD group (Fig. 3I–N) and not among NMOSD patients treated with rituximab. IL-2, IL-10, and IL-13 showed no characteristic pattern according to disease, therapeutic intervention, nor immunocomplex/complement stimulations. Detailed values of all cytokines are given in the Supplementary Table 3.

### AQP4-immunocomplexes and complement stimulation enhances CD16 expression in NK/NKT cells

**Figure 4:**
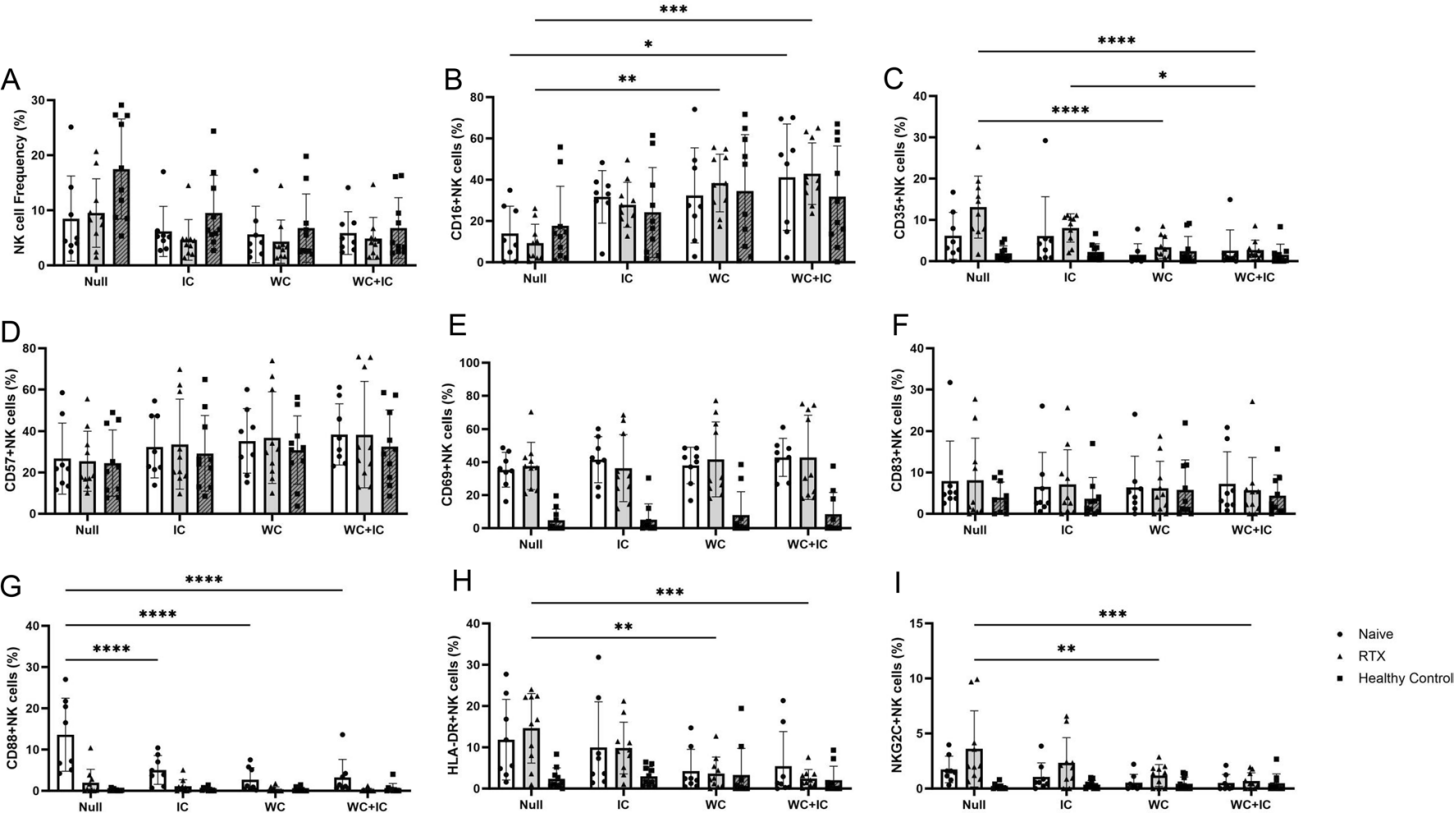
Flow cytometry analysis of NK cells after AQP4-immunocomplex- and whole complement-stimulation in vitro. The frequency of NK cells among total live lymphocytes (**A**) is shown. After extracting the CD3-/CD56dim + bright subset, CD16-positive (**B**), CD35-positive (**C**), CD57-positive (**D**), CD69-positive (**E**), CD83-positive (**F**), CD88-positive (**C**), HLA-DR-positive (**H**), and NKG2C-positive (**I**) subsets were gated. The stimulation with AQP4-immunocomplexes and whole complement caused CD16 upregulation in NK cells. Activation markers CD69 and CD83 showed no changes regardless of the stimulations. On the other hand, complement receptors CD35 and CD88 were downregulated by the stimulations. *IC* AQP4-immunocomplexes, *WC* whole complement, *WC* + *IC* AQP4-immunocomplexes/whole complement treated, *RTX* rituximab-treated NMOSD patients. NMOSD-Naive (n = 8), NMOSD-rituximab treated (n = 10), and healthy controls (n = 10). *p < 0.05; **p < 0.01; ***p < 0.001; ****p < 0.0001.

**Figure 5:**
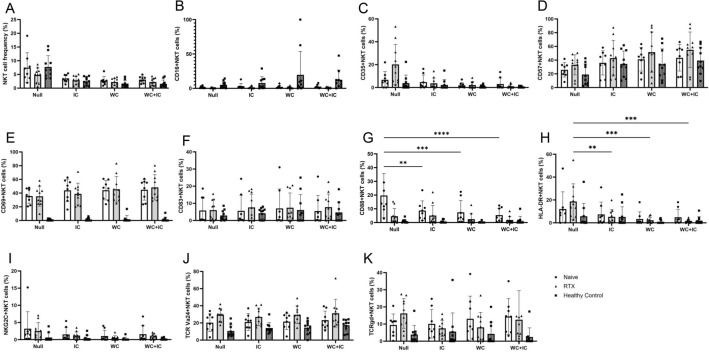
Flow cytometry analysis of NKT cells after AQP4-immunocomplex- and whole complement-stimulation in vitro. The frequency of NKT cells among total live lymphocytes (**A**) is shown. After extracting the CD3- and CD56- double positive subset, CD16-positive (**B**), CD35-positive (**C**), CD57-positive (**D**), CD69-positive (**E**), CD83-positive (**F**), CD88-positive (**C**), HLA-DR-positive (**H**), NKG2C-positive (**I**), TCR Vα24-positive (**J**), and TCR γδ-positive (**K**) NKT cells were analyzed. As well as NK cells, C5a receptor CD88 expression on NKT cells was downregulated by the stimulation with AQP4-immunocomplexes and whole complement. The activation markers analysis could not detect significant changes by the stimulations. *IC* AQP4-immunocomplexes, *WC* whole complement, *WC* + *IC* AQP4-immunocomplexes/whole complement treated, *RTX* rituximab-treated NMOSD patients. NMOSD-Naive (n = 8), NMOSD-rituximab treated (n = 10), and healthy controls (n = 10). *p < 0.05; **p < 0.01; ***p < 0.001; ****p < 0.0001.

After 24 h of AQP4-immunocomplex/whole complement stimulation, the PBMCs were also analyzed by flow cytometry (Figs. 4 and 5). The percentage of NK cells decreased in both NMOSD groups regardless of intervention. The percentage of NKT cells tended to decrease with the addition of the AQP4-immunocomplex or total complement component as noted previously^8^. However, among those remaining NK cells, CD16 expression levels increased with AQP4-immunocomplex/complement intervention, and was more pronounced in the NMOSD group. NKT cells in the NMOSD group did not show this CD16-expression response. Other cell surface markers showed no significant changes after stimulation with AQP4-immunocomplexes and all mixture of complement components. Detailed values of the flow cytometry analysis are provided in the Supplementary Table 4.

### NKT cells with follicular helper phenotype are increased in untreated NMOSD

**Figure 6:**
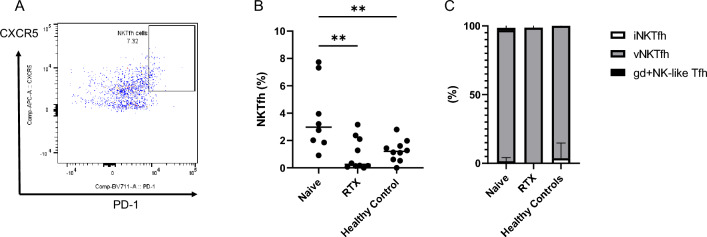
Follicular helper NKT cells in NMOSD. PD-1-positive CXCR5-positive subsets were extracted from CD3 + CD56 + CD11b-CD14-CD20-CD66b-NKT cells (**A**). The subset, NKT cells with follicular helper function (NKTfh) among lymphocytes, was significantly elevated in the untreated NMOSD group (**B**), and almost all NKTfh cells were TCR-Va24-negative/TCRγδ-negative variant NKT cells (**C**). *iNKTfh* invariant NKT cells with follicular helper function, *vNKTfh* variant NKT cells with follicular helper function, *gd* + *NK-like Tfh* TCRγδ-positive NK-like T cells with follicular helper function, *HC* healthy controls, *RTX* rituximab-treated patients. NMOSD-Naive (n = 8), NMOSD-rituximab treated (n = 10), and Healthy Controls (n = 10). *p < 0.05; **p < 0.01; ***p < 0.001; ****p < 0.0001.

Based on the above experiments, we measured the PD-1-positive CXCR5-positive subset, a hallmark of follicular helper T and NKT cells (Fig. 6A). Notably, follicular helper NKT cells among lymphocytes were significantly increased in the treatment naïve-NMOSD group as compared to RTX-treated NMOSD or HC (Naïve: 3.719 ± 2.526%; RTX: 0.9779 ± 1.168%; HC: 1.246 ± 0.7912%; P = 0.0023, Fig. 6B). A more detailed analysis of the subset revealed that almost all PD-1-positive CXCR5-positive cells were TCR Vα24-negative TCRγδ-negative variant NKT cells (Naïve: 95.33 ± 4.138%; RTX: 98.72 ± 3.039%; HC: 96.30 ± 11.10%) (Fig. 6C).

### Principal component analysis (PCA)

Factors from the flow cytometry and ELISA were included as input variables for PCA. Two analyses were performed separately: (i) the factors from all the variables, and (ii) the factors after AQP4-immunocomplexes and the mixture of all complement components stimulation. In the analysis using all the values as variables analyzed, we differentiated NMOSD patients from healthy controls by IL-8 values predominantly, followed by proinflammatory cytokines such as IL-6, TNFα, and IL-1β (Supplementary Fig. 2A). Similar plots were performed for the therapeutic intervention but could not differentiate adequately (Supplementary Fig 2B). Next, we performed the same analysis using only the AQP4 immunocomplex and complement stimulation groups as parameters. Here, we were able to more clearly distinguish NMOSD patients from healthy controls (Supplementary Fig. 2C). In the analysis, Principal Component 1 (PC1) was affected by IL-8, IL-23, TNFα, IL-27, CD69-positive NK/NKT cells, IL-6, IL-4, and IL-1β. In contrast, HLA-DR-positive NK/NKT cells and CD35/CD88-positive NKT cells accounted for a large proportion of Principal Component 2 (PC2). When plotted based on the therapeutic intervention, PC1 and PC2 tended to be higher in the Naive-NMOSD group (Supplementary Fig. 2D). Detailed PC1 and PC2 data analyzed by PCA are shown in Supplementary Tables 5 and 6.

## Discussion

**Figure 7:**
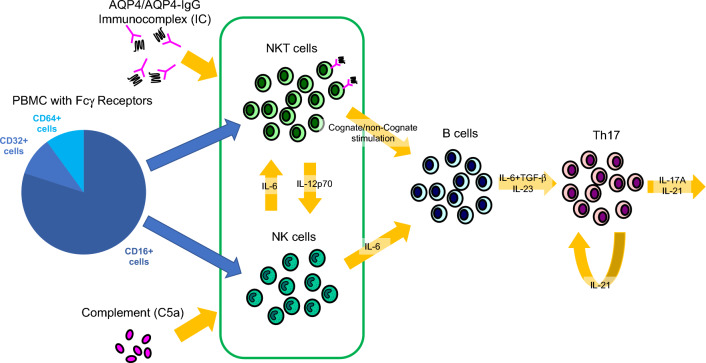
Potential pathway of AQP4 autoimmunity in NMOSD. AQP4-IgG binds to AQP4 protein to form AQP4-immunocomplexes. These bind primarily to CD16A on NKT cells. Simultaneously, granulocytes, monocytes/macrophages, and NK/NKT cells activated by complement (especially C5a) produce IL-6. These stimulated NKT cells to differentiate into NKTfh cells with follicular helper functions, and cognate stimulation activates B cells. In addition to IL-6, CD4 + T cells stimulated by IL-23 produced by B cells differentiate into Th17, which may exacerbate the autoimmune inflammatory cascade via IL-17A and IL-21 production.

In this study, we examined the effect of the AQP4-immunocomplex in the context of activated complement on PBMC phenotype and cytokine expression ex vivo. The AQP4-immunocomplex predominantly targeted variant NKT cells, which have diverse T-cell receptors (TCRs). Subsequently, these specialized NKT cells developed a follicular helper cell phenotype which was significantly enhanced in treatment-naïve patients as compared to patient treatment with RTX or HC individuals. Within 24 h of AQP4-immunocomplex and the mixture of all complement components stimulation, these cells elaborated pro-inflammatory cytokines including IL-6 and TNFα in all people with NMOSD. Specifically in treatment-naïve people with NMOSD, Th17 cytokines including IL-17A, -22, and -23 were also produced to a significantly greater extent than comparative cohorts. This pattern of cytokine production implies that T and B cells activated by IL-6 are necessary for downstream IL-17A/-22/-23 production (Fig. 7).

An essential fact in binding AQP4-immunocomplexes to PBMCs is that commercially available AQP4 antibodies labeled with fluorescent proteins recognize the intracellular domain of the AQP4 protein. This means that even if a nonspecific IgG recognizing the intracellular domain of AQP4 protein binds to PBMCs by chance, the signal cannot be detected by downstream flow cytometry in this experimental model. In addition, the use of AQP4 protein combined with pooled control IgG virtually eliminates all possibilities of (1) nonspecific binding between control IgG and AQP4 protein, (2) AQP4 protein itself binds to the cell surface without immunocomplexes, (3) endogenous AQP4-immunocomplexes are present in PBMC derived from NMOSD patients, and (4) pre-incubation binding of AQP4 immunocomplexes to NMOSD-PBMCs.

A Th17 cytokine, IL-23, is mainly produced by activated dendritic cells, macrophages, and monocytes. Memory B cells are also known to secrete IL-23 via B cell receptor signaling^11^. A report has indicated IL-23 elevation in the cerebrospinal fluid of NMOSD patients, along with IL-6, TGF-b1, and IL-10^12^. In the rituximab-treated NMOSD group in our data, elimination of B cells suppressed IL-23 production, which may have resulted in IL-17A production inhibition. There is a case report that IL-17A is significantly elevated in cerebrospinal fluid during an NMOSD relapse^13^. IL-17A and other Th17 cytokines may be more specific to NMOSD pathogenesis compared with MS^14^. Similarly, IL-21 levels in cerebrospinal fluid are elevated in NMOSD and correlate with complement activity^15^. IL-21 promotes and maintains Th17 lineage differentiation via STAT3 through an autocrine mechanism^16^. It has been reported that IL-21 receptors are expressed on T cells, B cells, and NK cells, contributing to cell activation, differentiation, and proliferation^17^. In another report, IL-21 in plasma and cerebrospinal fluid was similarly elevated in NMOSD and decreased with therapeutic intervention^18^. IL-21 may be essential in differentiating switched memory B cells into antibody-producing cells^19^ and IL-21 also enhances antibody production in an antigen-independent manner^20^ supporting its important role in our proposed model in the inflammatory pathway of AQP4 autoimmunity (Fig. 7).

Principal Component Analysis revealed that IL-8 was the top factor separating the NMOSD group from healthy controls. While a comprehensive analysis of cytokine levels in cerebrospinal fluid of NMOSD patients showed that IL-8 was significantly elevated compared to multiple sclerosis and controls^21^, another study using AQP4-IgG-positive NMOSD sera showed no significant elevation between healthy controls^22^. IL-8 is known as a chemotactic factor for granulocytes, mainly neutrophils, and also functions as a promoter of phagocytosis induction and angiogenesis^23^. In the pathogenesis of NMOSD, a mechanism has been proposed in which neutrophils activated by the complement degradant C5a induce astrocyte injury via glutamate toxicity^24^, suggesting that AQP4-immunocomplex stimulation may exacerbate this pathological response.

In contrast to the marked changes in cytokine production, flow cytometry analysis of PBMCs, especially NK/NKT cells, after AQP4 immunocomplex and complement stimulation showed no meaningful changes in cell surface markers, including complement receptors and activation markers. Firstly, this could be due to the short stimulation period. While this study used 24-h stimulation, more prolonged stimulation may have been necessary to induce changes in cell surface markers. Second, the ex vivo environment of the culture may not simulate the events occurring in the NMOSD patient.

There is ongoing inconsistency in the literature about the prevalence of NK and NKT cells in NMOSD and other autoimmune diseases. Several previous reports have shown that the prevalence of NK/NKT cells and their CD16 expression are significantly decreased in NMOSD^5,8,25,26^. Our results here add to that finding by showing that the AQP4-immunocomplex with complement causes a further decrease of NK/NKT cells. The reduction of NK/NKT cells in NMOSD may be a common mechanism in autoimmune diseases that alters the ratio of regulatory and cytotoxic NK/NKT cells, leading to autoimmunity.

Recent research has shed light on the existence of a subset of NKT cells that possess characteristics reminiscent of Tfh cells, known as NKTfh cells^27^. NKTfh cells are a distinct subset of NKT cells that exhibit phenotypic and functional similarities to Tfh cells, characterized by the expression of surface markers such as programmed cell death protein 1 (PD-1), CXCR5, and ICOS. NKTfh cells also exhibit a distinct cytokine profile, with the production of IL-21^27^. NKTfh cells have been shown to provide help to B cells and promote the production of antigen-specific antibodies. Through their interaction with B cells, NKTfh cells can influence the selection of high-affinity B cells, class-switching, and the formation of long-lived plasma cells. Additionally, NKTfh cells have been implicated in autoimmune diseases and certain infections, highlighting their potential role in immune dysregulation and pathogenesis^28–30^.

Variant NKT cells, also known as Type II NKT cells, are more abundant in humans then invariant NKT cells^31^. This subset has a diverse TCR repertoire in response to lipid antigens and reacts to sulfatides rather than α-galactosyl ceramide. They can behave in a pathogenic or protective manner but their particular role remains elusive^31^. Our data in NMOSD specifically points to variant NKT cells, which may appear to be responding to the AQP4-immunocomplex by taking a follicular helper phenotype and producing pro-inflammatory cytokines that can orchestrate antigen-specific B cell differentiation and autoantibody production. Another subset of NKT cells corresponding to the TCR Vα24-negative TCR γδ-negative population are Mucosal-associated invariant T (MAIT) cells. MAIT cells are a population of cells that, like invariant NKT cells, have NK receptors but a different invariant TCR (TCR Vα7.2-Jα33). They represent 0.1–10% of human blood T cells and are involved in the pathogenesis of autoimmune diseases, infections, and malignancies^32,33^. Although MAIT cells in NMOSD are not clarified, the results obtained in this study suggest that MAIT cells may be involved in the pathogenesis of these diseases.

Based on these, we formulated a speculative model of NMOSD pathogenesis focused on the upstream inflammatory pathway for AQP4 autoimmunity (Fig. 7): AQP4-IgG and AQP4 protein-conjugating AQP4-immunocomplexes bind primarily to CD16A on NKT cells. Spurred by IL-6 and activated complement (especially C5a), NKT cells differentiate into NKTfh cells and activates B cells through cognate B cell stimulation. CD4 + T cells stimulated by IL-23 differentiate into pathogenic Th17, triggering a pro-inflammatory cycle via IL-17A and IL-21 production. This hypothesis may explain why the recently approved C5 inhibitors, IL-6 inhibitors, and B cell-depleting therapies for NMOSD have been so efficacious in preventing relapses.

There are several limitations to our present study. First, the analysis was performed using an artificial in vitro system, which may not accurately reproduce the events occurring in patients. More accurate evaluation of AQP4 autoimmunity may be facilitated by adding AQP4-immunocomplexes, complement, and specific cytokine stimuli to the system to simulate the phenomena occurring in the NMOSD patients. Second, the duration of stimulation used in the in vitro system was only 24 h. Although 24-h incubation is sufficient time to detect changes in cytokine release^34^, in vivo NMOSD pathology may involve much longer stimulation of AQP4-immunocomplexes and complement, and *ex viv*o culture might even have reversed the cell-surface markers. Longer time and artificial addition of inflammatory cytokines may provide further insight into cell surface markers that have shown little change in the study. Third, we used an in vitro system with AQP4 protein and AQP4-IgG-positive NMOSD patient-derived heat-inactivated serum. However, we did not verify whether the response was AQP4-specific or whether other CNS proteins (e.g. myelin oligodendrocyte glycoprotein: MOG) could also show similar responses. It is conceivable that differences in complement regulatory protein levels in distinct patient samples may influence PBMC activation and cytokine production in a manner that was not designed to be investigated in this study. Fourth, this study did not use TCR Vα7.2, a MAIT cell-specific marker, to isolate MAIT cells; the TCR Vα24-negative TCR γδ-negative population likely includes many MAIT cells. More detailed studies, including MAIT cells, are needed in the following study. Finally, the variant NKT cells identified in this study have no specific markers or methods to distinguish them from invariant NKT cells. Considering that variant NKT cells are still an enigmatic and heterogenous cell population, it is necessary to investigate how they transmit signals downstream after AQP4-immunocomplex binding, using methods such as analysis of single cells using next-generation sequencing. Fifth, this study does not directly demonstrate whether NKTfh is an AQP4-specific responder or how it activates B cells; a detailed mechanism of cognate or indirect activation on B cells would be expected in future studies.

In summary, the present study provides experimental support indicating that variant NKT cells are primary responders to the AQP4-IgG/AQP4 immunocomplex. In the presence of activated complement, this interaction appears to induce a strong Th17 cytokine bias, consistent with features of neuroinflammation characteristic of clinical NMOSD. NKTfh activities were significantly elevated in treatment-naive NMOSD patients and compared to those treated with rituximab, implicating a coordinated program of T and B cells along with AQP4-IgG and complement in pathogenesis of NMOSD. Together, these findings may offer new insights into AQP4 autoimmunity, and advance NKTfh as a potential new therapeutic target.

## Materials and methods

### Patients and peripheral blood mononuclear cells (PBMC)

PBMCs from serum AQP4-IgG positive NMOSD patients measured by cell-based assay (CBA) were donated from Prof. Friedemann Paul and The Guthy-Jackson Foundation. Eight naïve NMOSD samples were obtained before treatment intervention. Additionally, ten rituximab-treated NMOSD samples were collected from patients in remission who were at least 4 weeks from their last relapse. Healthy controls (HC) were obtained from the Guthy-Jackson Charitable Foundation and healthy volunteers under informed consent. The PBMCs were isolated by conventional Ficoll's method. The demographics of study participants are shown in Supplementary Table 1.

Pooled sera from 10 AQP4-IgG-seropositive-NMOSD patients were heat-inactivated at 56 °C for 30 min. One milligram of full-length human recombinant AQP4 protein (Abnova Taiwan, #H00000361-P01) per 10 mL of serum was added and cultured for 24 h in a rotator to form AQP4-immunocomplexes. A mixture of heat-inactivated healthy-control serum and AQP4 protein was used as a control group. AQP4-immunocomplexes were added to the culture medium at 10 μL per 0.1 M PBMC (Fig. 1A). For AQP4 uptake experiments, PBMCs were incubated under 5% CO_2_ at 37 °C for 1 or 8 h. Internalized AQP4-immunocomplexes are calculated by the difference between the AQP4-positive cells with and without 0.3% tween-20 permeabilization. 20% volume of Pooled Human Complement (Cedarlane Laboratories Ltd. #ICSER10ML) was used for PBMC stimulation. PBMCs were aliquoted at 0.1 M/100 μL culture medium per well and incubated for 24 h at 37 °C 5% CO_2_ using 96-well plates for the downstream cell surface marker and cytokine analysis.

### Flow cytometry and data analysis

Flow cytometry analysis was performed as the previous report^8^. Briefly, BD Fortessa X-20 (BD Bioscience) and Cytek® Aurora (Cytek) were used for the analysis. After doublet cells were excluded, lymphocytes and monocytes fractions were isolated by plotting forward- and side-scatter heights. The antibodies used for the assay are shown in Supplementary Table 2. Dead cells were excluded from analysis using LIVE/DEAD™ Fixable Blue Dead Cell Stain Kit (Thermo Fisher Scientific). After Fc receptor blocking using FcR Blocking Reagent (Immunostep), PBMCs were stained with the surface markers for 30 min at 4 °C. They were then fixed with 4% paraformaldehyde, permeabilized with 0.1% Tween-20. Cells were stained with intracellular markers for 30 min at 4 °C in PBS with 0.5% fetal bovine serum (FBS) and 2 mM of Ethylenediaminetetraacetic acid (EDTA). The AQP4-FITC, CXCR5-APC, and PD-1-BV711 gatings were performed using their isotype controls (Biorbyt #orb248103; BioLegend #400122; and BioLegend #400168, respectively). PBMC subsets were defined as follows: NK cells (CD45 + /CD14-/CD3-/CD56dim&bright), NKT cells (CD45 + /CD14-/CD3 + /CD56dim&bright), T cells (CD45 + /CD14-/CD3 + /CD56-), NKTfh cells (CD45 + /CD14-/CD3 + /CD56dim&bright/CXCR5 + /PD-1 +), iNKT cells (CD45 + /CD14-/CD3 + /CD56dim&bright/TCR Vα24 + /TCR γδ–), vNKT cells (CD45 + /CD14-/CD3 + /CD56dim&bright/TCR Vα24-/TCR γδ–), TCRγδ + NK-like T cells (CD45 + /CD14-/CD3 + /CD56dim&bright/TCR Vα24-/TCR γδ +). The gating strategy in the study is shown in Supplementary Fig. 1.

### Multiple enzyme-linked immunosorbent assay (ELISA)

V-PLEX® Proinflammatory Panel 1 Human Kit and Th17 Panel 1 Human Kit (Meso Scale Diagnostics) were used for the cytokine detection according to the manufacturer’s instruction. IFN-γ, IL-1β, IL-2, IL-4, IL-6, IL-8, IL-10, IL-12p70, IL-13, TNF-α, IL-17A, IL-21, IL-22, IL-23, IL-27, IL-31, and MIP-3α levels were analyzed using the supernatant of 24 h-incubated PBMCs. The MESO QuickPlex SQ 120MM (Meso Scale Diagnostics) was used for data acquisition.

### Statistical analysis

Data were analyzed with FlowJo v10.7.1 (Becton Dickinson & Company) and GraphPad Prism 9.5.1 (GraphPad Software, LLC). The groups were compared using the two-way ANOVA, and Spearman’s rank correlation was used for the analysis of correlations between parameters. Due to the exploratory nature of the study no adjustment for multiple comparisons was made. We also performed principal component analysis (PCA) by GraphPad Prism 9.5.1, to determine what factors contribute to the diagnosis and therapeutic intervention. Principal components (PCs) were selected based on the two-largest eigenvalues. A statistical significance was defined as P < 0.05.

### Ethics approval and consent to participate

All of the experiments were conducted under IRB approval from Massachusetts General Hospital, protocol number 2019P003556. All methods were performed in accordance with the relevant guidelines and regulations. All participants provided informed consent for data and specimen drawing for research at registry sites sponsored by the Massachusetts General Hospital, Guthy-Jackson Charitable Foundation, and at Charité-Universitätsmedizin Berlin under an existing IRB approval.

### Supplementary Information

Supplementary Table 1.

Supplementary Table 2.

Supplementary Table 3.

Supplementary Table 4.

Supplementary Table 5.

Supplementary Table 6.

Supplementary Legends.

Supplementary Figures.

## Data Availability

The data supporting this study’s findings are available from the corresponding authors, [SN, LM], upon reasonable request.

## Abbreviations

ADCC: Antibody-dependent cellular cytotoxicity
AQP4: Aquaporin-4
Anti-AQP4-IgG: Anti-aquaporin-4-IgG
APC: Antigen-presenting cells
CNS: Central nervous system
CSF: Cerebrospinal fluid
DC: Disease controls
GFAP: Glial fibrillary acidic protein
Ig: Immunoglobulin
iNKT cells: Invariant NKT cells
MOG: Myelin oligodendrocyte glycoprotein
anti-MOG-ab: Anti myelin oligodendrocyte glycoprotein antibody
MS: Multiple sclerosis
NC: Normal controls
NK cells: Natural killer cells
NKT cells: Natural killer-T cells
NMOSD: Neuromyelitis optica spectrum disorder
PBMC: Peripheral blood mononuclear cells
RTX: Rituximab
vNKT cells: Variant NKT cells
