# Supplementary material for: Anti-aquaporin-4 immune complex stimulates complement-dependent Th17 cytokine release in neuromyelitis optica spectrum disorders
Source: Sci Rep. 2024 Feb 7;14:3146. doi: 10.1038/s41598-024-53661-5 (PMC10850367; doi:10.1038/s41598-024-53661-5)
Supplement: Supplementary file 7 — Supplementary Legends. [file 41598_2024_53661_MOESM7_ESM.docx]

**Supplemental Figure and Table Legends.**

**Supplemental Figure 1: The gating strategy of flow cytometry in the study.**

(A) After extracting singlets and lymphocytes by forward and side scatter plots, dead cells and CD45-negative cells were excluded. NK cells were defined as CD3-/CD56dim+bright subsets, and NKT cells were defined as CD3+/CD56+ subsets. CD16/CD35/CD88/CD69/CD83/CD57/NKG2C/HLA-DR-positive cell were analyzed. (B) PBMCs from NMOSD patients and Healthy Controls were incubated with AQP4-immunocomplexes. AQP4-FITC-positive cells were gated from Live/CD45+ cells after forward and side scatter plots.

**Supplemental Figure 2: The Principal Component Analysis (PCA) from ELISA- and Flow cytometry-derived parameters.**

PCA was performed using all the parameters in the study (A and B), or the parameters from AQP4-immunocomplexes/complement stimulations (C and D). Red dots are from NMOSD patients (n=18), and black dots are from Healthy Controls (n=10) in the diagnosis plot (A and C). Similarly, red dots are for Naïve-NMOSD (n=8), blue dots for Rituximab-treated-NMOSD (n=10), and black dots for Healthy Controls (n=10) in the treatment intervention plot (B and D). The parameters from AQP4-immunocomplexes/complement stimulations allowed a more apparent contrast between the two groups (C).

**Supplemental Table 1: Profiles of each group in the cohort**.

Age, sex, and AQP4-IgG serostatus of this cohort's NMOSD patient and control groups.

**Supplemental Table 2: Flow cytometry panels in the study.**

Details of the type, clone, manufacturer, and production ID of the fluorescent antibodies for flow cytometry used in this study.

**Supplemental Table 3: Cytokine levels after AQP4-immunocomplexes/complement stimulation analyzed by multiple-ELISA kits.**

Detailed cytokine levels in the culture medium under each condition were analyzed using the Multiple ELISA kit.

IC: AQP4-immunocomplexes; WC: whole complement; WCIC: AQP4-immunocomplexes/whole complement treated; RTX: Rituximab-treated NMOSD patients. NMOSD-Naive (n=8), NMOSD-rituximab treated (n=10), and Healthy Controls (n=10).

**Supplemental Table 4: Cell surface marker expressions after AQP4-immunocomplexes/complement stimulation analyzed by flow cytometry.**

Subset detail values for each antigen-positive NK/NKT cell calculated by flow cytometry.

IC: AQP4-immunocomplexes; WC: whole complement; WCIC: AQP4-immunocomplexes/whole complement treated; RTX: Rituximab-treated NMOSD patients. NMOSD-Naive (n=8), NMOSD-rituximab treated (n=10), and Healthy Controls (n=10).

**Supplemental Table 5: Detailed PC1/PC2 data obtained by PCA based on all parameters.**

**Supplemental Table 6: Detailed PC1/PC2 data obtained by PCA based on the parameters after AQP-immunocomplexes/complement stimulation.**

Detailed PC1 and PC2 values in PCA, based on parameters obtained in this study using flow cytometry and ELISA, including all stimulation conditions (Sup. Table 5) and after AQP4-immunocomplex/complement stimulation (Sup. Table 6). Proinflammatory cytokines, Th17 cytokines, and activated NK/NKT cells are high in PC1, suggesting that these factors are essential in differentiating NMOSD patients from healthy controls.

IC: AQP4-immunocomplexes; WC: whole complement; WC+IC: AQP4-immunocomplexes/whole complement treated; RTX: Rituximab-treated NMOSD patients. NMOSD-Naive (n=8), NMOSD-rituximab treated (n=10), and Healthy Controls (n=10).
