# Supplementary material for: Anti-aquaporin-4 immune complex stimulates complement-dependent Th17 cytokine release in neuromyelitis optica spectrum disorders
Source: Sci Rep. 2024 Feb 7;14:3146. doi: 10.1038/s41598-024-53661-5 (PMC10850367; doi:10.1038/s41598-024-53661-5)
Supplement: Supplementary file 8 — Supplementary Figures. [file 41598_2024_53661_MOESM8_ESM.pptx]

## Slide 1
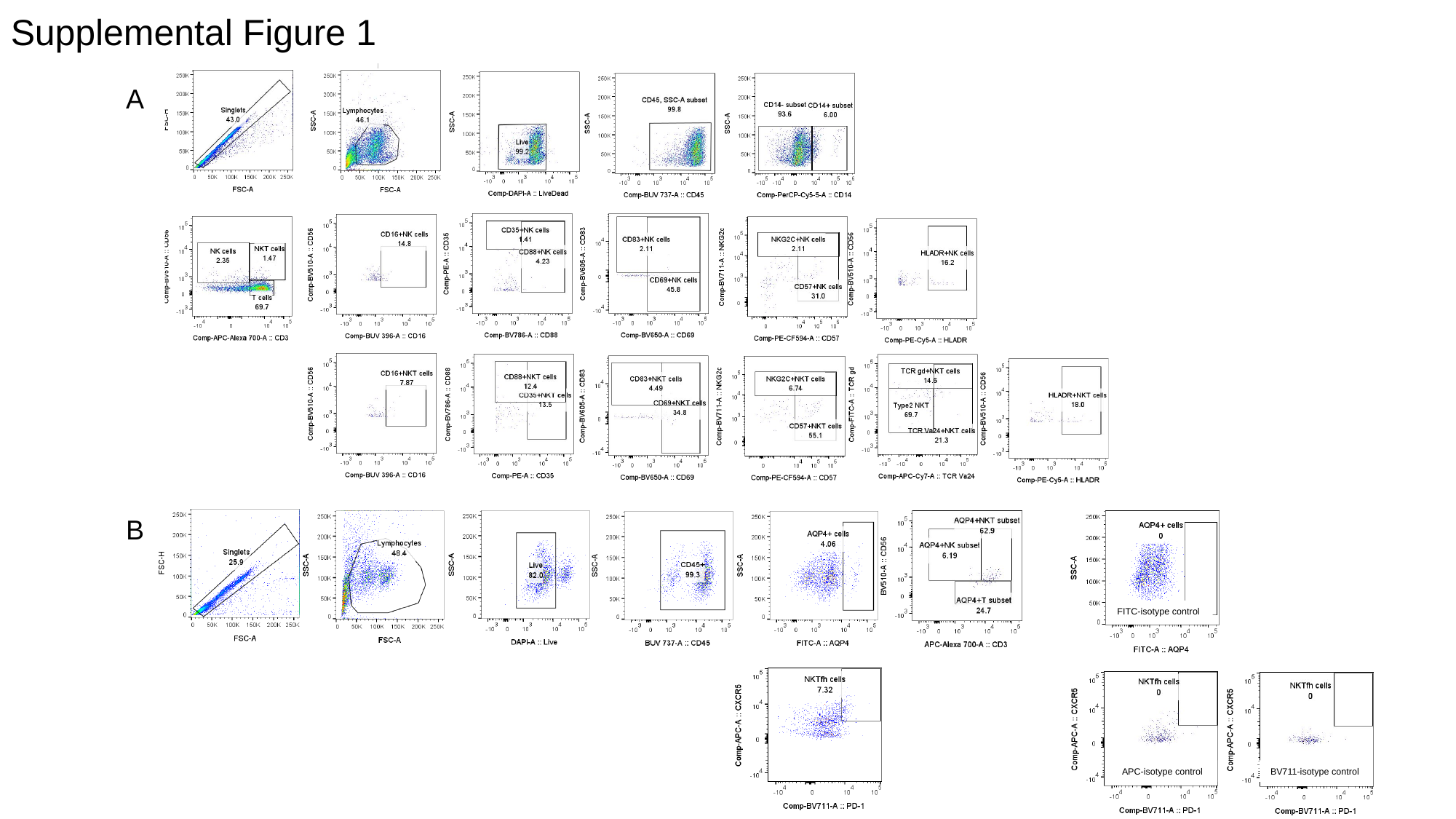

Supplemental Figure 1
A
B
FITC-isotype control
APC-isotype control
BV711-isotype control

## Slide 2
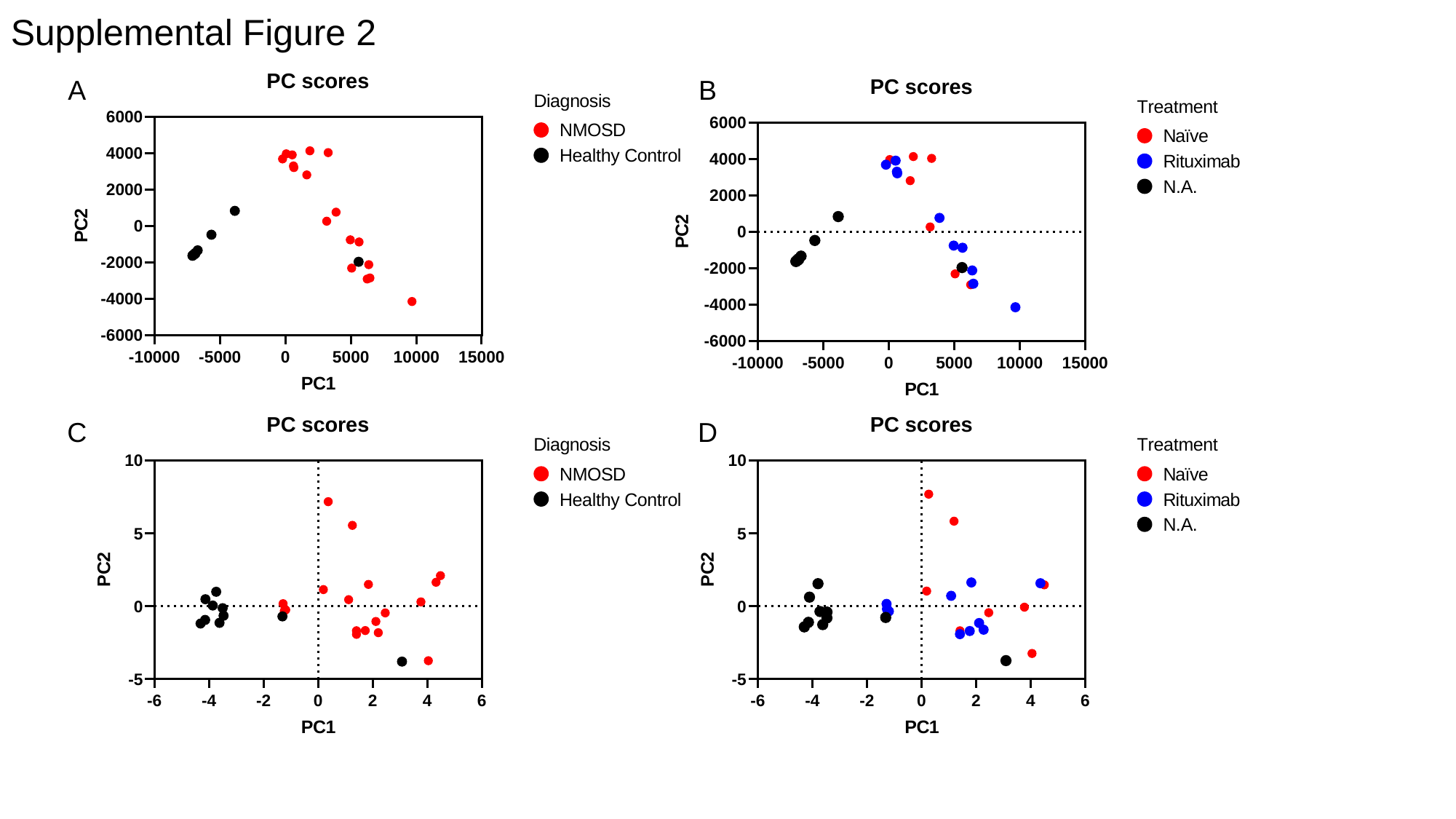

Supplemental Figure 2
A
B
C
D
